# The association of hypertension among married Indian couples: a nationally representative cross-sectional study

**DOI:** 10.21203/rs.3.rs-3865512/v1

**Published:** 2024-02-02

**Authors:** Jithin Sam Varghese, Arpita Ghosh, Aryeh Stein, KM Venkat Narayan, Shivani Patel

## Abstract

Mounting evidence demonstrates that intimate partners sharing risk factors have similar propensities for chronic conditions such as hypertension. The objective was to study whether spousal hypertension was associated with one’s own hypertension status independent of known risk factors, and stratified by socio-demographic subgroups (age, sex, wealth quintile, caste endogamy). Data were from heterosexual married couples (n = 50,023, women: 18-49y, men: 21-54y) who participated in the National Family Health Survey-V (2019-21). Hypertension was defined as self-reported diagnosis of hypertension or average of three blood pressure measurements ≥ 140 systolic or 90 mmHg diastolic BP. Among married adults, the prevalence of hypertension among men (38.8 years [SD: 8.3]) and women (33.9 years [SD: 7.9]) were 29.1% [95%CI: 28.5–29.8] and 20.6% [95%CI: 20.0-21.1] respectively. The prevalence of hypertension among both partners was 8.4% [95%CI: 8.0-8.8]. Women and men were more likely to have hypertension if their spouses had the condition (husband with hypertension: PR = 1.37 [95%CI: 1.30–1.44]; wife with hypertension: PR = 1.32 [95%CI: 1.26–1.38]), after adjusting for known risk factors. Spouse’s hypertension status was consistently associated with own status across all socio-demographic subgroups examined. These findings present opportunities to consider married couples as a unit in efforts to diagnose and treat hypertension.

## Introduction

India has the second largest population with hypertension in the world of over 220 million people [1]. Although current national guidelines recommend opportunistic screening of all adults and universal screening for those 30 years and older, only 2 in 5 with hypertension are diagnosed [2–7]. Married couples often share chronic disease status, a phenomenon referred to in the literature as spousal concordance, which is only partly explained by concordance of individual risk factors [8–10]. Limited evidence is available on spousal concordance in global populations, among whom chronic disease are rapidly rising and for whom public health infrastructure for screening and prevention of disease is limited. Elucidating the magnitude of spousal concordance in such settings may suggest whether couple-based strategies for hypertension detection and prevention would improve upon societally- and individually-focused strategies [11, 12].

India also stands to benefit from the investigation of spousal concordance because of the local epidemiology of hypertension. Studies suggest an earlier onset of cardiometabolic disease among Indians, relative to other populations [13]. This puts India's reproductive-age population, which is the second largest in the world after China's, at risk of catastrophic health expenditures and declining economic productivity [14]. Therefore, whether spousal concordance exists after accounting for socio-demographic determinants (e.g., age, education, household size), health risks (e.g., body mass index, alcohol, and tobacco use) and environmental features (e.g., urbanicity) can present opportunities to better understand modifiable mechanisms underlying shared hypertension risk among married couples. Specifically, evaluating whether the presence of hypertension in one spouse could be leveraged to identify hypertension in the other spouse in the reproductive-age population is also intriguing. We investigated spousal concordance in hypertension in India using nationally representative data from married adults surveyed in 2019–2021 (n = 50,023).

## Methods

### Study Population

The National Family Health Survey-5 (NFHS-5) was a stratified, two-stage probability proportional to size sample survey of 724,115 women and 101,839 men from 636,699 households. The survey was conducted in two phases, from June 2019 to March 2020 and November 2020 to April 2021. From this sample, 57,693 heterosexual couples of reproductive age were identified, of which 54,356 were of legal age at marriage (men: 21 years, women: 18 years), defined as men aged 21–54 years ("husbands") and women aged 18–49 years ("wives") who participated in NFHS-5. We further restricted the analytic sample to the 50,023 couples who had data on key variables of interest for hypertension and in which the woman was not currently pregnant. A flowchart of the analytic sample is provided in **Supplementary Fig. 1**. This study was deemed exempt from review by the Institutional Review Board of Emory University because it was a secondary data analysis of de-identified data.

### Hypertension status

Hypertension was defined as composite of measured blood pressure above thresholds defined by the Indian Council of Medical Research [2] and self-reported history of hypertension. Among adults without a reported history of hypertension, we averaged the three values of systolic blood pressure (SBP) and diastolic blood pressure (DBP) measured using OMRON Blood Pressure monitors in the participants' homes [15]. We then classified hypertension as having SBP greater than or equal to 140 mm Hg or greater than or DBP equal to 90 mm Hg [2]. All adults reporting a history of diagnosed hypertension (i.e., those responding "yes" response to "Do you currently have hypertension?" or "Before this survey, were you ever told you had high blood pressure by a doctor, nurse, or health practitioner on two or more occasions?") were classified as having hypertension. Medication usage was not considered in the definition because it was asked only of those who self-reported a diagnosis of hypertension. We defined undiagnosed hypertension as proportions of adults with hypertension who self-reported not being diagnosed prior to the survey.

### Individual and household characteristics

Individual and household covariates known to be associated with hypertension were included based on availability in the analytic sample [16]. Individual-level covariates of interest were educational attainment (primary or up to 4th class, secondary or up to 10th class, post-secondary), body mass index (BMI) derived from measured height and weight (underweight: BMI $< 18.5$ kg/m$^2$, normal: BMI: $18.5 \leq$ BMI $<$ 25.0 kg/m$^2$, overweight or obese: BMI $>$ 25.0 kg/m$^2$), alcohol consumption (yes or no) and smoking status (yes or no). These were available for men and women separately. Weight and height were measured using standardized protocols with SECA 874 U digital scale and SECA 213 Stadiometer. BMI

was defined as weight (in kg) divided by height (in square meters). Household covariates of interest were number of children, size of household, rural residence (versus urban) and wealth quintile (relative wealth within each state). Social caste membership, a measure of class and caste endogamy, was assessed following categories provided by the NFHS and used by the Government of India. Based on self-report, couples were classified as either both General, both Other Backward Castes (OBC), both Schedule Caste/Scheduled Tribe, and Inter-caste.

## Statistical Analysis

All analyses accounted for the complex multi-stage survey design and clustering at the level of primary sampling units, using survey weights provided by Demographic and Health Surveys [17].

## Estimation of prevalence of co-occurrent hypertension among spouses

We defined spousal concordance as co-occurrence of status in both partners for hypertension. Although concordance is typically defined as both partners having the same status (hypertension or no hypertension), we used this definition to be consistent with the prior literature on spousal concordance. To evaluate whether the prevalence of disease co-occurrence was beyond what is statistically expected under assumptions of independence, we also report the product of the marginal prevalence in husbands and wives. To understand whether concordant status could be used to detect hypertension, we attributed the proportion of undiagnosed hypertension in married men and women by the hypertension status (no hypertension, diagnosed, undiagnosed) of their spouse.

## Association of spouse’s hypertension status with own hypertension status

Analyses were carried out separately for husbands and for wives. We estimated the prevalence ratio (PR) and 95% CI describing the concordance of hypertension (exponentiating $\beta_{11}$ and $\beta_{21}$) between spouses using survey-weighted Poisson regression with robust standard errors. Models were specified as follows:

*log [Pr(Husband’s Hypertension = 1|Covariates)] =* $\beta_{10} + \beta_{11}$ *Wife’s Hypertension + Husband’s Individual Covariates + Shared Household Covariates* (Eq. 1**a)**

*log [Pr(Wife’s Hypertension = 1|Covariates)] =* $\beta_{20} + \beta_{21}$ *Husband’s Hypertension + Wife’s Individual Covariates + Shared Household Covariates* (Eq. 1**b)**

We present estimates adjusted for socio-demographic determinants (age, education, social caste background), health risks (body mass index, alcohol, and tobacco use) and environmental features (urbanicity). The models were also adjusted for state fixed effects. The first set of models were estimated for the nation. Subsequently, we evaluated associations between spouses stratified by the individual’s age (< 40 vs ≥ 40), individual’s attained level of education, rurality, household wealth and caste endogamy groups. Associations by socio-demographic subgroups were estimated by modifying

Equations 1a and 1b to include an interaction term between the spouse's disease and an indicator for the subgroup level.

### Imputation of missing data

We used multiple imputation with predictive mean matching (10 datasets, 50 iterations) under a missing at random assumption and included auxiliary individual (weight, height, body mass index, blood pressure, random glucose, waist circumference, years of education, status of diagnosis and medication use for diabetes and for hypertension) and household covariates (caste and religion) [18]. We pooled all associations using Rubin's rules for imputed datasets.

### Sensitivity Analyses

To assess the robustness of our results for unmeasured shared characteristics that are correlated with the spouse's health behaviors and socio-economic position, we repeated the main analysis after adjusting for characteristics of the spouse of the index participant (**Supplementary Fig. 2**). To increase study power, we estimated the association using systolic blood pressure as a continuous variable. Previous studies have reported differences in spousal concordance by sex, given differences in marital experience and health behaviors [19]. Therefore, we estimated if there were sex differences in magnitude of association between spouses using a pooled dataset and an interaction term of spouse's disease status and individual's sex (male = 1, female = 0). We used generalized estimating equations under a Poisson distribution with logarithmic link and robust standard errors to account for couple-level clustering of observations. We added binary indicator variables for husbands (yes = 1, no = 0). We assessed sex differences in spousal concordance based on the magnitude and confidence intervals of the interaction term. We did not conduct a pooled test with and without the interaction term since likelihood based methods are not applicable to generalized estimating equations [20]. To assess robustness of results for selection bias in participation for blood pressure data collection, we imputed the hypertension status of 4,333 couples using multiple imputation and repeated the main analysis.

All analysis was carried out using R v4.2.0 and statistical packages (survey 4.1.1, geepack 1.3.9).

## Results

### Descriptive characteristics of husbands and wives

Descriptive characteristics of the 50,023 married couples are provided in Table 1. Husbands were older than wives (mean age 38.8 [SD: 8.3] and 33.9 [SD: 7.9] years, respectively) and had attained more years of schooling (mean years 7.9 [SD: 4.9] and 6.7 [SD: 5.2], respectively). Tobacco and alcohol use were higher among husbands (tobacco: 52.0%, alcohol: 29.2%) compared to wives (tobacco: 5.4%, alcohol: 1.1%). Husbands were more likely to have overweight but not obesity (husbands: 24.2% vs wives: 20.6%) but wives were more likely to have obesity (husbands: 5.0%, wives: 7.4%). The crude prevalence of hypertension was higher among husbands (29.1% [95%CI: 28.5, 29.8]) than wives (20.6% [95%CI: 20.0, 21.1]). Respondents excluded from the present analysis were similar to the analytic sample

(**Supplementary Table 1**). Missingness in analytic sample was under 0.5% for all variables (**Supplementary Table 2**).

Table 1
Descriptive characteristics of married couples in India, 2019-21

| | **Analytic sample (n = 50,023)** | |
|---|---|---|
| | **Husbands** | **Wives** |
| **Individual characteristics** | | |
| Age in years | 38.8 (38.7, 38.9) | 33.9 (33.8, 34.0) |
| Years of schooling | 7.9 (7.8, 8) | 6.7 (6.6, 6.8) |
| Consumes alcohol (%) | 29.2 (28.4, 30.0) | 1.1 (0.9, 1.2) |
| Tobacco use (%) | 52.0 (51.2, 52.8) | 5.4 (5.1, 5.7) |
| Body mass index (kg/m$^2$) | 23.3 (23.3, 23.4) | 23.1 (23.0, 23.1) |
| **Weight status** | | |
| Underweight (< 18.5kg/m$^2$) | 8.9 (8.5, 9.3) | 13.9 (13.5, 14.4) |
| Normal (18.5–24.9kg/m$^2$) | 61.6 (60.9, 62.3) | 57.8 (57.1, 58.6) |
| Overweight (25-29.9kg/m$^2$) | 24.2 (23.5, 24.8) | 20.6 (20.1, 21.2) |
| Obese (≥ 30 kg/m$^2$) | 5.0 (4.7, 5.3) | 7.4 (7, 7.8) |
| **Attained schooling** | | |
| No education | 16 (15.4, 16.5) | 27.5 (26.8, 28.2) |
| Primary | 15.9 (15.4, 16.5) | 14.2 (13.8, 14.7) |
| Secondary | 52.9 (52.2, 53.6) | 46.3 (45.6, 47) |
| Higher | 15.2 (14.5, 15.9) | 12 (11.4, 12.6) |
| **Household characteristics** | | |
| Number of children | 5.5 (5.4, 5.5) | |
| Household members | 2.3 (2.3, 2.4) | |
| **Household wealth quintile at national level (%)** | | |
| Q1 | 18.1 (17.4, 18.9) | |
| Q2 | 20.1 (19.4, 20.7) | |
| Q3 | 21.2 (20.6, 21.9) | |

Data are from the NFHS V. All values are survey weighted means (for continuous variables) or percentages (for categorical variables) and 95% confidence intervals for the estimates.

| | Analytic sample (n = 50,023) | |
|---|---|---|
| | **Husbands** | **Wives** |
| **Individual characteristics** | | |
| Q4 | 21 (20.3, 21.7) | |
| Q5 | 19.6 (18.6, 20.5) | |
| **Rural (%)** | 69 (67.4, 70.5) | |
| **Caste endogamy (%)** | | |
| Inter-caste | 16.9 (16.3, 17.5) | |
| Both General | 20 (19.2, 20.9) | |
| Both Other Backward Castes | 36.9 (36, 37.8) | |
| Both Scheduled Caste/Tribe | 26.1 (25.2, 27.1) | |
| **Chronic disease outcomes** | | |
| Systolic Blood Pressure (mm Hg) | 125.4 (125.2, 125.6) | 117.6 (117.4, 117.8) |
| Diastolic Blood Pressure (mm Hg) | 82.9 (82.8, 83.1) | 79.1 (79.0, 79.2) |
| Hypertension (%) | 29.1 (28.5, 29.8) | 20.6 (20.0, 21.1) |
| Self-reported Hypertension (%) | 9.1 (8.6, 9.5) | 10.8 (10.3, 11.2) |
| Co-occurring Hypertension (%) | 8.4 (8.0, 8.8) | |

Data are from the NFHS V. All values are survey weighted means (for continuous variables) or percentages (for categorical variables) and 95% confidence intervals for the estimates.

## The prevalence of concordant hypertension among spouses

The prevalence of concordant (co-occurrent) hypertension in husbands and wives was 8.4% (95%CI: 8.0, 8.8), whereas the expected prevalence was 6.0%. State-level prevalence of observed concordant hypertension is provided in Fig. 1. Prevalence of concordant hypertension varied between states. State-level prevalence of hypertension among husbands and wives are provided in **Supplementary Fig. 3**, with states in South and East India displaying higher prevalence.

Among those with hypertension, 52.4% (95%CI: 50.8, 54.0) women and 31.1% (95%CI: 29.9, 32.4) men were previously diagnosed. Among married adults with undiagnosed hypertension (**Supplementary Fig. 4**), 10.1% (95%CI: 8.8, 11.6) of women and 10.2% (95%CI: 9.4, 11.1) of men had a spouse with diagnosed hypertension, 28.9% (95%CI: 27.1, 30.8) of women and 14.1% (95%CI: 13.1, 15.2) of men had a spouse with undiagnosed hypertension, and 61.0% (95%CI: 58.8, 63.1) of women and 75.7% (95%CI: 74.4, 76.9) of men had a spouse without hypertension.

## Association of hypertension status between spouses in the Indian population

We observed positive associations between the disease status of husbands and wives and their spouses for hypertension beyond that attributable to both individual and household risk factors. Men married to women with hypertension had 1.32 (95%CI: 1.26, 1.38) times higher prevalence of hypertension, while women married to men with hypertension had 1.37 (95%CI: 1.30, 1.44) times higher prevalence of hypertension (Table 2). Associations of individual and household risk factors with hypertension are presented in **Supplementary Table 4**.

Table 2
Associations of hypertension status between couples for wives and husbands in India, 2019-21

| Prevalence Ratio (95% CI) | Husbands | Wives |
|---|---|---|
| Adjusted for individual and household risk factors | 1.32 (1.26, 1.38) | 1.37 (1.30, 1.44) |
| Additionally adjusted for spousal risk factors | 1.31 (1.25, 1.37) | 1.38 (1.31, 1.45) |

Associations for Husbands and Wives are the association of spouse's hypertension status with own hypertension status. All models adjusted for individual (age, schooling, body mass index, number of children, alcohol and tobacco use), and household (household members, number of children, rurality, wealth quintile) factors and state fixed-effects.

Spouse's disease status was consistently and positively associated with own hypertension status, respectively, in all age, education, urbanicity, wealth and caste endogamy groups examined (Fig. 2). A higher magnitude of spousal concordance of hypertension among wives were observed when the wives' age were less than 40 years (Age < 40: 1.55 [95%CI: 1.44, 1.66] vs ≥ 40: 1.28 [95%CI: 1.20, 1.37]).

## Sensitivity Analysis

The overall results did not change when models were further adjusted for spouse's additional characteristics apart from hypertension (Table 2; **Supplementary Table 5**) in addition to one's own personal characteristics and household characteristics. Spouse's SBP was positively associated with own SBP (husbands' SBP: 0.12 [95%CI: 0.11, 0.13] mmHg per mmHg; wives' SBP: 0.13 [95%CI: 0.11, 0.14 (**Supplementary Table 6**). Spousal concordance was also similar in the study population who were multiply imputed before excluding those without data on hypertension (**Supplementary Table 7**). The magnitude of spousal concordance in hypertension was also similar by sex. The statistical interaction term in magnitude of spousal concordance between husbands and wives did not suggest differences by sex for hypertension (0.94 [95%CI: 0.88, 1.01]).

## Discussion

In this nationally representative investigation of spousal chronic disease concordance in India, we observed that adults married to individuals with hypertension were more likely to have these conditions themselves, even after accounting for several established predictors of hypertension. Positive associations between spouses in hypertension prevalence were observed across rural and urban areas, wealth strata, and several other demographic characteristics. Magnitude of spousal concordance did not vary by sex. Finally, spousal concordance was stronger when an individual's age was less than 40 years or households were in the bottom 20% of the wealth distribution.

Consistent with a recent study of spousal concordance of hypertension in India among middle-aged and older couples (co-occurrence: 19.8%, prevalence ratio: 1.19 [95%CI: 1.15–1.24]), we observed positive association of hypertension status among younger couples [23]. In a study of 1,598 spousal dyads from 4 sites across India, the magnitude of spousal concordance in hypertension was very similar to what we found here. The relative odds of hypertension was 1.20 times higher in wives whose husbands had hypertension, although the association was not statistically significant [24]. A meta-analysis of 8 studies from Brazil, China, Russia, United Kingdom, and the USA, comprising 81,928 spouse pairs (20–94 years) also concluded that being married to someone with hypertension was associated with higher odds (1.41, 95% CI: 1.21–1.64) of hypertension [9]. Our study adds to the literature by being larger than any one of these studies.

Spousal concordance was consistently stronger in instances in groups with lower frequency of the outcome—such as comparing concordance in younger versus older couples or poorest versus richest couples. The stronger prevalence ratio at lower marginal prevalence of the outcome is expected statistically. This is because the prevalence ratio as an estimate of spousal concordance, when assessed separately for husbands (Eq. 1a) and wives (Eq. 1b), would approach 1 as the marginal prevalence approaches 100%. Moreover, a greater difference between observed and expected joint prevalence is required to achieve the same magnitude of spousal concordance as that at a lower prevalence. For further comparison of our approach to alternate approaches to assess spousal concordance, we refer the reader to **Supplementary Note 1**.

Several mechanisms for spousal concordance in chronic disease status have been proposed. Individuals tend to marry those who are like them in terms of social class, ethnicity and health behaviors. The convergence hypothesis suggests that once individuals marry, their health behaviors become more concordant over time, possibly through interpersonal influences on health behaviors as well as shared influences of common environments after marriage. India's marital demography makes it an informative setting to study this phenomenon. In India, most of the population marries within the same social caste, ethnic and class groups, a form of socially structured assortative mating. Caste, ethnic, and class membership often dictate dietary and lifestyle choices [25–27]. This provides for clustering of disease development due to shared genetic predisposition and behavioral risk factors that exist both before and after marriage. Furthermore, Indians marry at younger ages and divorce at lower rates compared to populations in high-income nations, and thus have earlier and longer opportunities to exert influence on the health of their spouse across the life course. Beyond assortative mating and health convergence, physiological stress responses to relationship quality [28], intimate partner violence [29], and spouse's emotions [30], may be pathways through which marital relationships affect health. For example, spousal support for physical activity, beyond self-monitoring and evaluation, was a mediator of a successful short-term physical activity intervention among Swiss couples [31]. While we were not able to evaluate mechanisms for spousal concordance in this cross-sectional study, the finding that concordance was observed across socio-economic and urban-rural spectrums suggests that factors leading to spousal concordance transcend demographic groups. Moreover, we observed similar spousal concordance among Inter-caste and same-caste couples, suggesting consanguinity and similar early life environments are not the sole drivers of spousal concordance.

Spousal concordance in the prevalence of hypertension, with greater co-occurrence than what is statistically expected, suggests that couple- or family-centered interventions may be useful for improving screening and diagnosis efforts, especially since over half of hypertension in India remains undiagnosed [5–7]. Such an approach may improve the efficiency of screening, since guidelines presently incorporate only family history of first-degree relatives (mother, father, sister, brother), but not that of other residents of the household (spouse, children) when screening for disease [32]. Other studies have also highlighted the potential for couple-centered interventions for management of chronic disease after diagnosis, although the effectiveness of such strategies in Indian contexts are unknown [11].

Although this study is nationally representative and probably the largest of its kind, there are some limitations. First, NFHS is limited to adults of reproductive age, who are younger than most at-risk individuals. Therefore, the findings are not generalizable to older adults, among whom prevalence of hypertension in India is as high as 45.9% [4]. Second, prior diagnosis of hypertension was based on self-report, and field assessments at one time point are subject to information bias and measurement error. Third, we were unable to account for duration of marriage or spousal concordance in behavioral risk factors such as dietary intake and physical activity, since these factors were unavailable. Balancing these limitations, use of this dataset allowed us to estimate spousal concordance by demographic characteristics while accounting for several individual- and household-characteristics that are established risk factors for chronic disease.

In conclusion, we provide robust evidence of spousal concordance as a relevant and pervasive phenomenon in the burden of hypertension in India. There is a need for longitudinal studies that would facilitate partitioning of risk between shared genetics and shared environments before and after marriage, to better identify mechanisms of concordance that are amenable to intervention. Such determinants may include early life undernutrition experienced by communities or exposure to pollutants [33, 34]. However, regardless of mechanisms driving shared spousal risks for hypertension, these data present actionable opportunities for innovative screening strategies, and potential family-based interventions, that target at-risk couples and families to achieve timely detection and treatment of hypertension.

## Declarations

**Sources of Support:** This study was supported by the National Heart, Lung and Blood Institute (P01HL154996 to KMVN and SAP) and National Institute of Diabetes and Digestive and Kidney Disorders (P30DK111024 to KMVN) of the National Institutes of Health.

**Conflict of interest:** Authors declare no conflicts of interest.

**Previous Presentation:** A preliminary version of the data in this article was presented at American Diabetes Association's Scientific Sessions 2023 (San Diego, CA)

**ORCID**: Jithin Sam Varghese (0000-0003-3825-9897), Aryeh D. Stein (0000-0003-1138-6458), K.M. Venkat Narayan (0000-0001-8621-5405), Shivani A. Patel (0000-0003-0082-5857)

**Disclosures:** Authors declare no conflicts of interest.

**Author contributions**: SAP, AG, ADS and JSV conceptualized the study and the analysis plan. JSV conducted the analysis and wrote the first draft with inputs from SAP. All authors edited subsequent drafts and approved the final draft.

**Acknowledgements:** We thank the participants, survey enumerators and coordinators of the National Family Health Survey 2019-21.

**Data Availability Statement:** The datasets are free to download after approval from www.dhsprogram.com. The code for the analysis is available on https://github.com/jvargh7/nfhs5_couples.

## Figures

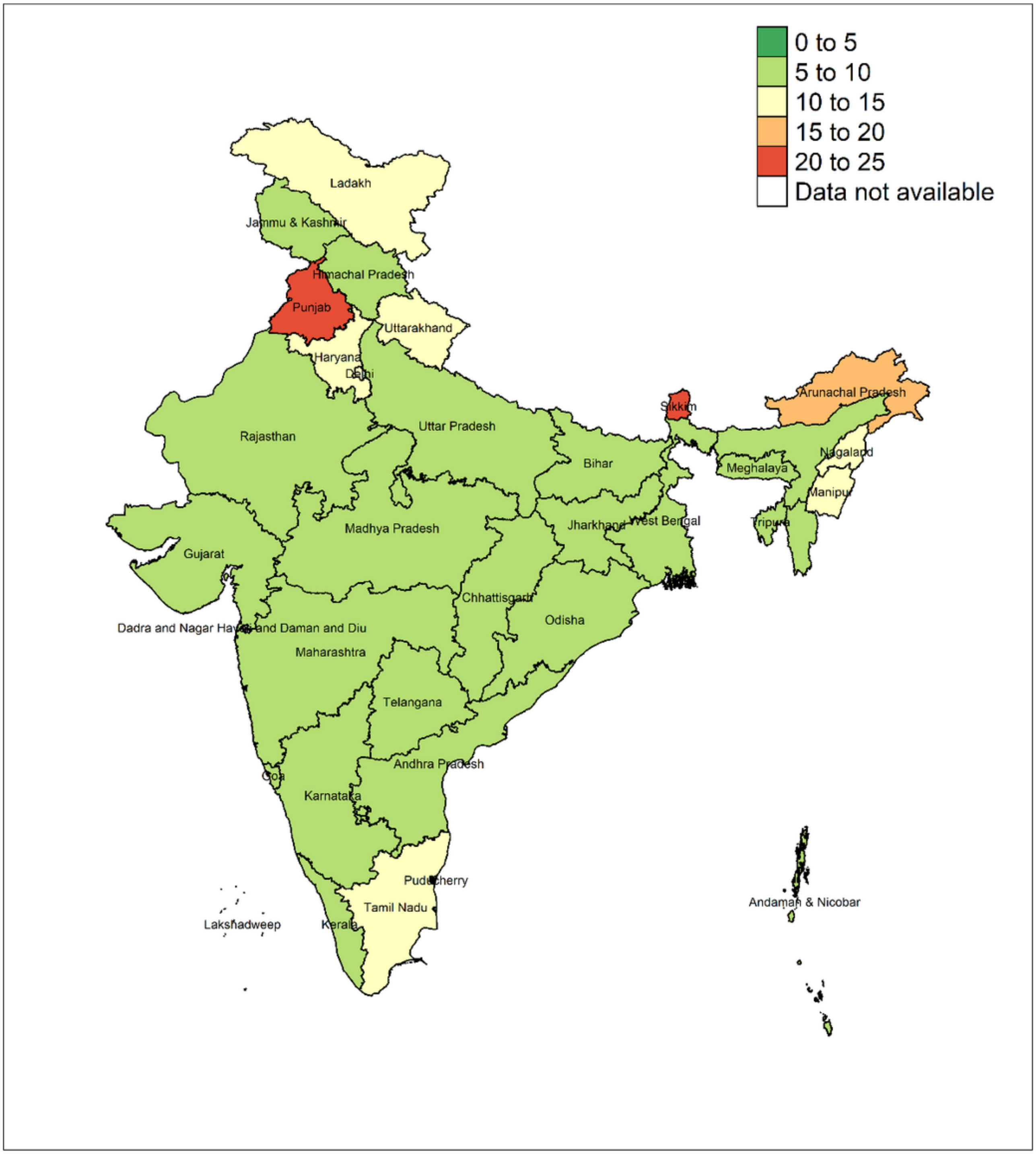

### Figure 1

#### Prevalence of co-occurrent hypertension in married Indian couples, n = 50,023

Co-occurrent hypertension was defined as both spouses having the respective disease. The marginal and co-occurrent prevalence of disease are provided in **Supplementary Table 3**.

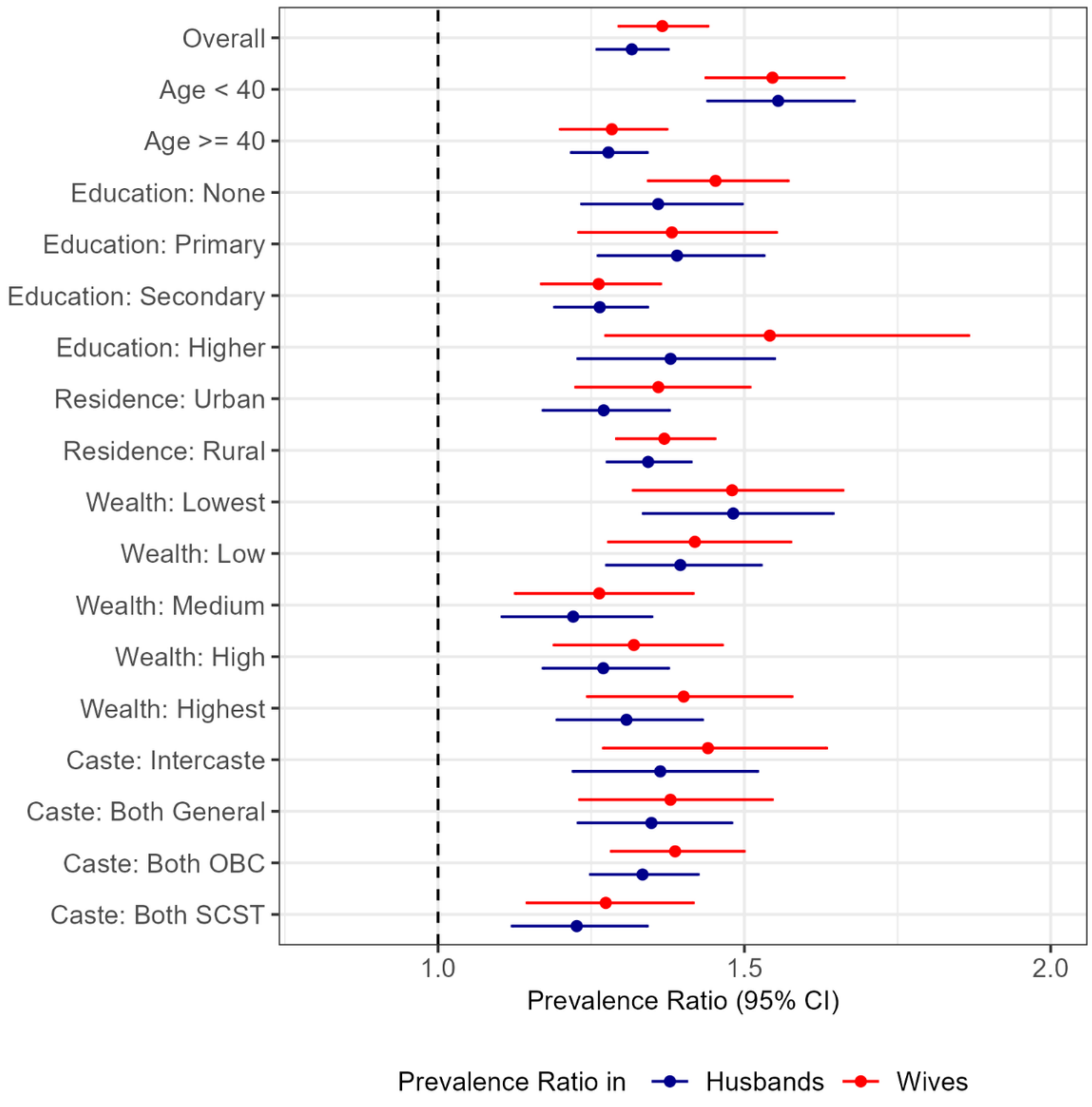

**Figure 2**

**Stratum-specific prevalence ratio by socio-demographic groups, n = 50,023**

Association of spousal disease status with own disease status for Hypertension: diagnosed or SBP/DBP ≥140/90 mmHg. Estimates are available in **Supplementary Table 2**.

All models adjusted for individual (age, schooling, body mass index, number of children, alcohol and tobacco use), and household (household members, number of children, rurality, wealth quintile) factors and state fixed-effects.

### Supplementary Files

This is a list of supplementary files associated with this preprint. Click to download.

- SupplementaryMaterial20240110.docx
- STROBESpousalconcordance20240110.doc
